# Validation of stroke volume and cardiac output by electrical interrogation of the brachial artery in normals: assessment of strengths, limitations, and sources of error

**DOI:** 10.1007/s10877-015-9668-9

**Published:** 2015-02-15

**Authors:** Donald P. Bernstein, Isaac C. Henry, Harry J. Lemmens, Janell L. Chaltas, Anthony N. DeMaria, James B. Moon, Andrew M. Kahn

**Affiliations:** 1 Sotera Wireless, Inc., 10020 Huennekens Street, San Diego, CA 92121 USA; 2 Department of Medicine, University of California San Diego School of Medicine, San Diego, CA 92103 USA; 3 Department of Anesthesia, Stanford University School of Medicine, Stanford, CA 94305-5115 USA

**Keywords:** Stroke volume, Cardiac output, Noninvasive, Transbrachial electrical velocimetry, Impedance cardiography

## Abstract

The goal of this study is to validate a new, continuous, noninvasive stroke volume (SV) method, known as transbrachial electrical bioimpedance velocimetry (TBEV). TBEV SV was compared to SV obtained by cardiac magnetic resonance imaging (cMRI) in normal humans devoid of clinically apparent heart disease. Thirty-two (32) volunteers were enrolled in the study. Each subject was evaluated by echocardiography to assure that no aortic or mitral valve disease was present. Subsequently, each subject underwent electrical interrogation of the brachial artery by means of a high frequency, low amplitude alternating current. A first TBEV SV estimate was obtained. Immediately after the initial TBEV study, subjects underwent cMRI, using steady-state precession imaging to obtain a volumetric estimate of SV. Following cMRI, the TBEV SV study was repeated. Comparing the cMRI-derived SV to that of TBEV, the two TBEV estimates were averaged and compared to the cMRI standard. CO was computed as the product of SV and heart rate. Statistical methods consisted of Bland–Altman and linear regression analysis. TBEV SV and CO estimates were obtained in 30 of the 32 subjects enrolled. Bland–Altman analysis of pre- and post-cMRI TBEV SV showed a mean bias of 2.87 % (2.05 mL), precision of 13.59 % (11.99 mL) and 95 % limits of agreement (LOA) of +29.51 % (25.55 mL) and −23.77 % (−21.45 mL). Regression analysis for pre- and post-cMRI TBEV SV values yielded y = 0.76x + 25.1 and r^2^ = 0.71 (r = 0.84). Bland–Altman analysis comparing cMRI SV with averaged TBEV SV showed a mean bias of −1.56 % (−1.53 mL), precision of 13.47 % (12.84 mL), 95 % LOA of +24.85 % (+23.64 mL) and −27.97 % (−26.7 mL) and percent error = 26.2 %. For correlation analysis, the regression equation was y = 0.82x + 19.1 and correlation coefficient r^2^ = 0.61 (r = 0.78). Bland–Altman analysis of averaged pre- and post-cMRI TBEV CO versus cMRI CO yielded a mean bias of 5.01 % (0.32 L min^−1^), precision of 12.85 % (0.77 L min^−1^), 95 % LOA of +30.20 % (+0.1.83 L min^−1^) and −20.7 % (−1.19 L min^−1^) and percent error = 24.8 %. Regression analysis yielded y = 0.92x + 0.78, correlation coefficient r^2^ = 0.74 (r = 0.86). TBEV is a novel, noninvasive method, which provides satisfactory estimates of SV and CO in normal humans.

## Introduction

Ejection of SV is the primary mechanical function of the heart and, in response to metabolic oxygen requirements SV is modulated by contractility, loading conditions, and heart rate to produce the requisite cardiac output (CO). CO is the ultimate expression of cardiovascular performance and, because it is the cornerstone of oxygen transport goal-oriented therapy, its assessment and optimization in critically-ill humans is of paramount importance [1]. Thermodilution cardiac output (TDCO) is currently the gold standard method for CO determination in the critically ill [2, 3]. However, with the virtual demise of the pulmonary artery catheter as a diagnostic tool for estimating left ventricular preload [4, 5], its justification and indications for TDCO determination are becoming slimmer and more stringent [6, 7]. Because of its invasiveness and potential complications, a search for minimally invasive or noninvasive alternatives for TDCO is ongoing [8–10].

What is readily apparent from review of the literature is the paucity of accurate, simple, technician-independent, “hands-off”, continuous, noninvasive CO methods, which do not require external calibration [3, 8–10]. Transthoracic electrical bioimpedance cardiography, otherwise known as impedance cardiography (ICG), is a technology that fulfills all the requirements for an ideal CO method, except for its lack of consistent accuracy [3, 8–11]. Because of the generally poor performance of the transthoracic technique in sick humans [11, 12], and especially those with excess intrathoracic extravascular lung water [13, 14], the technology has been deemed of marginal usefulness in the care of the critically ill [11].

This study in normal humans concerns the validation of a new noninvasive, velocimetric-based, pulsatile SV technique, known as transbrachial electrical bioimpedance velocimetry (TBEV) [15]. As compared to ICG implemented transthoracically, TBEV is, in theory and method, an ICG equivalent, but operationally signal acquisition is obtained from the upper arm. For this validation study, TBEV SV is compared with that obtained from cardiac magnetic resonance imaging (cMRI), which is considered the absolute “gold-standard” for determination of ventricular volumes [16, 17].

## Methods

### Study population

Thirty-two (32) volunteer adult subjects were recruited for the study. By history, physical examination, electrocardiography, echocardiography, and magnetic resonance imaging, the subjects showed no demonstrable evidence of heart disease. Since cMRI is considered the absolute gold standard for measurement of ventricular volumes [16, 17], SV obtained from that technique was compared to SV determined by TBEV [15]. In order to obtain a wide spectrum of SV and CO, subjects were recruited with a broad range of heights, weights, and body mass indices (BMI). Body surface area (BSA) for each subject was calculated using the Mosteller equation [18] (Table 1). This study was approved by the Institutional Review Board of the University of California, as well as Aspire Institutional Review Board, San Diego, and all subjects provided written and oral informed consent.

**Table 1:** Subject characteristics and cMRI measurements: all data are presented as the mean ± standard deviation (SD)

Males (%)	24 (75 %)	End diastolic volume	154 ± 26.9 mL
Age	41.5 ± 12.5 years	End systolic volume	58.1 ± 11.9 mL
Weight	83.3 ± 20.2 kg	Stroke volume	96.1 ± 18.6 mL
Body mass index	26 ± 4.4 kg m^−2^	Ejection fraction	62.2 ± 4.6 %
Body surface area	2.02 ± 0.30 m^2^	Cardiac output	6.08 ± 1.3 L min^−1^
Heart rate	63.3 ± 7.6 beats min^−1^	Cardiac index	3.00 ± 0.41 L min^−1^ m^−2^

See text (Sect. 2.4) for calculation of SV and ejection fraction

### Echocardiography protocol

All subjects underwent echocardiography, including color, pulsed, and continuous wave Doppler. Studies were performed using a commercially available echograph (iE33, Philips Medical, Andover, MA) with a broadband 2.0–3.5 MHz phased-array transducer to assess the presence of mitral and/or aortic valve disease. This was to ensure that all participants had structurally normal hearts. Subjects were positioned in the left lateral decubitus position and standard images were obtained from the parasternal and apical windows in accordance with current guidelines [19]. Data were acquired and stored in digital format, then analyzed offline using a separate workstation.

### MRI protocol

Subjects were scanned on a Signa 1.5T HDx scanner (GE Healthcare, Milwaukee, WI). After localizing images were obtained, a short axis stack of images through the entire left ventricle was acquired during end-expiration, using steady-state free precession (SSFP) imaging with a slice thickness of 8 mm, spacing of 2 mm, in-plane grid of 224 × 224, and acceleration factor of 1.5. The number of views per segment was adjusted as needed to yield a temporal resolution of 50 ms or less, and the field of view was adjusted as needed, based upon each subject’s habitus. HR was recorded for each acquisition and the average HR during imaging was computed. Each cMRI scan was completed in approximately 1 (one) hour.

### cMRI image analysis

MRI images were analyzed offline, using an Advantage Workstation with ReportCard 4.0 (GE Healthcare, Milwaukee, WI). End-systolic and end-diastolic frames were manually identified by visual inspection of the largest and smallest volume. Then, for each slice, the endocardium was manually traced at these two time points, including the papillary muscles with the contours, and left ventricular volume was subsequently calculated using the modified Simpson’s method [16]. SV was determined as the difference between the end-diastolic and end-systolic volumes. CO was then calculated as the product of measured SV and the average recorded HR. Stroke index (SI) and cardiac index (CI) were calculated as the SV and CO divided by BSA.

### Transbrachial electrical (bioimpedance) velocimetry (TBEV)

#### TBEV device

**Fig. 1:**
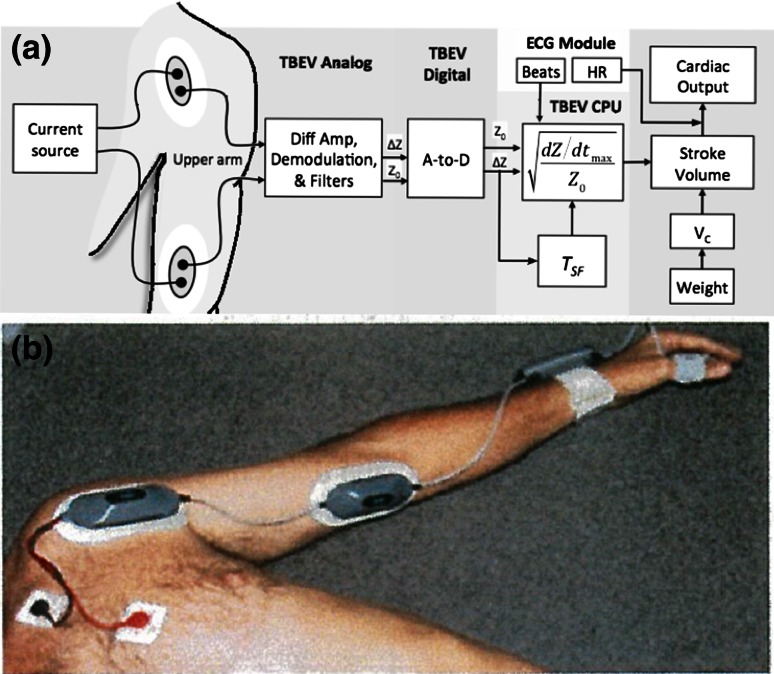
**a** A pictorial circuit diagram from which SV and CO are obtained. Shown are the upper and lower housings for the electrode patches on the arm. AC is injected via the outer electrodes and voltage electrodes are shown as the inner electrodes. Voltage is demodulated, amplified and filtered to obtain Z_0_ and ∆Z(t), which then undergo analog to digital (A to D) conversion. Electronic differentiation of ∆Z(t) yields dZ/dt. dZ/dt_max_/Z_0_ undergoes square root transformation. T_SF_ (systolic flow time) is multiplied by the volume conductor (V_C_) and ([dZ/dt_max_/Z_0_])^0.5^ to obtain stroke volume (SV). SV is multiplied by heart rate (HR) to obtain cardiac output (CO). Details found in text. **b** Photograph of volunteer with ECG patch electrodes on upper chest, TBEV electrode housing on the upper brachium, and wiring to an electrode housing just proximal to the antecubital fossa. The impedance signals are relayed to a signal processor shown on the wrist. Also shown is a pulse oximeter on thumb

The TBEV system is a wearable, battery-powered, four-lead impedance cardiograph. It is worn on the upper arm and is held in place only by custom adhesive electrode patches (Vermed^®^, Inc, Bellows Falls, VT, USA) (Fig. 1a). The system consists of two electrical housings connected by an electric cable: (1) a proximal assembly that is attached to a dual-element electrode, which is placed below the clavicle, proximal to the humeral head and axilla; and (2) a dual-element distal assembly which is attached to the anteromedial surface of the upper arm, proximal to the antecubital fossa (Fig. 1a, b). Using this electrode arrangement for interrogation of the brachial artery, an alternating current (ac, I, A) field is applied along and within the long axis of the upper arm. As a result of passing ac through an electrical impedance Z (ohm, Ω) (i.e. resistance to ac), a potential difference (voltage, U) within the current field is obtained. This is an expression of Ohm’s Law applied to biological tissues (i.e. IZ = U). The proximal assembly houses the upper voltage sensor and current driver, and the distal assembly houses the electronic circuit board into which the lower voltage sensor and current driver are integrated [15]. Figure 1b is a photograph of a human volunteer wearing the TBEV montage.

**Fig. 2:**
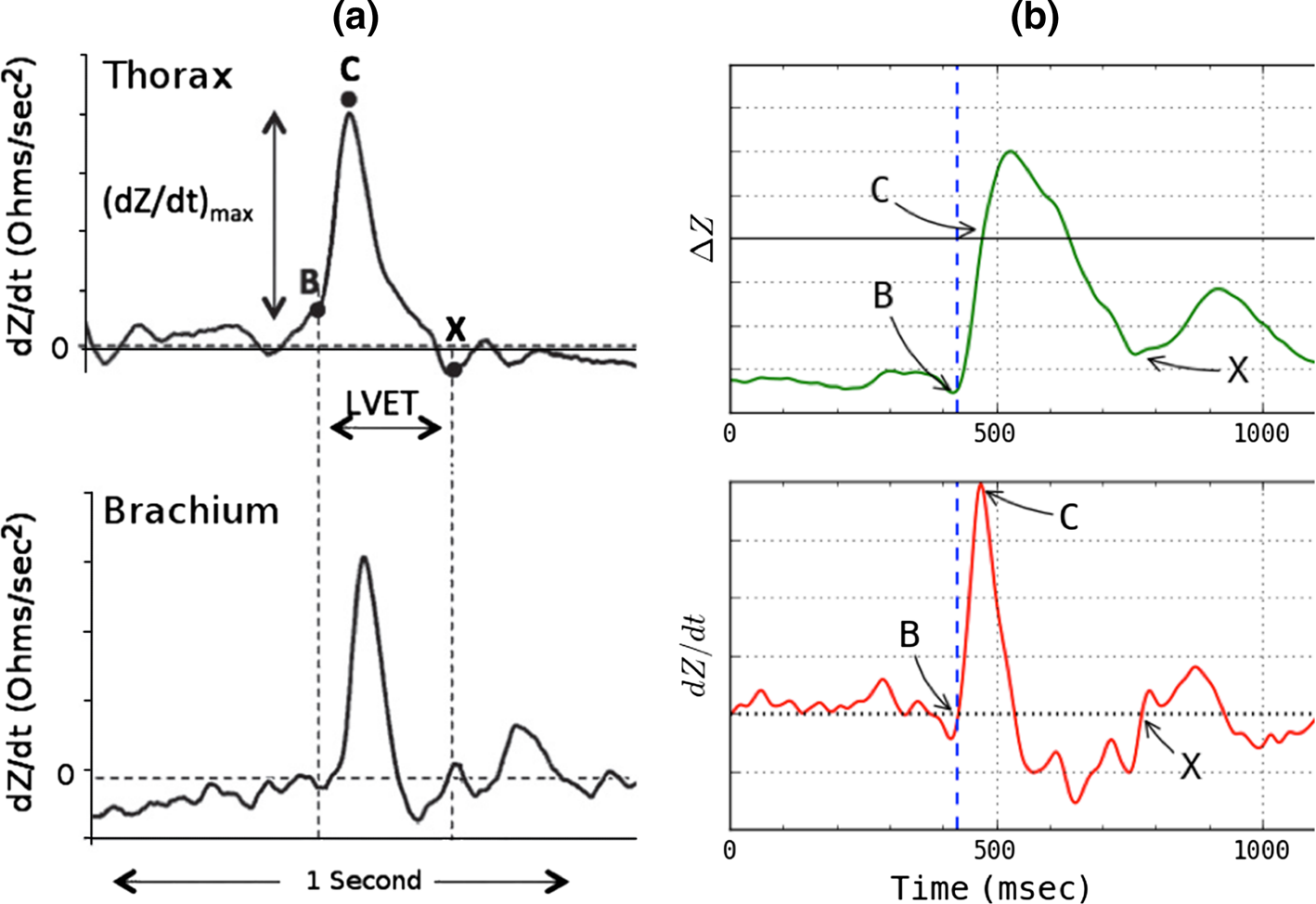
**a** In the upper tracing is shown a dZ/dt waveform from the thorax. In the lower tracing is shown a dZ/dt tracing from the brachium. Note that for the transthoracic waveform, the end of ejection (flow) is the nadir after the first zero crossing after point C, dZ/dt_max_. In the lower tracing is shown a dZ/dt waveform obtained from the brachium. Note that the end of flow is the second zero crossing after point C, dZ/dt_max_. The waveforms were obtained simultaneously, but, because of the transmission delay, they were aligned synchronously in time to illustrate the same T_SF_. **b** The transbrachial impedance pulse variation ∆Z (*upper tracing*) and its differentiated first time-derivative dZ/dt (*lower tracing*). In both tracings the onset of flow is marked as point B, the maximum forward slope of ∆Z and peak first time-derivative dZ/dt_max_ as point C, and termination of flow as point X [20]. The temporal interval from B to X is systolic flow time T_SF_

The TBEV analog circuit emits a constant magnitude, small amplitude 70 kHz, 4 mA RMS ac source, driving the signal through a segment of the upper arm through the current driver electrodes (Fig. 1a). The 70 kHz carrier signals from each voltage sensor first pass through identical filters (25 kHz low pass and 20 kHz high pass) and amplification stages. The signals are then differentially amplified and passed to an amplitude demodulation circuit. The high and low frequency components of the total transthoracic impedance (Z(t)) are then separated into two major components; (1) ∆Z, further comprising (a), a sinusoidal respiratory component, which is filtered out, and (b), a superimposed pulsatile time-variable ac cardiodynamic component (∆Z(t)), and (2) Z_0_, a quasi-static transbrachial base impedance, respectively. Further signal processing of ΔZ(t) entails finding the peak value of the first time-derivative of dZ/dt (i.e. dZ/dt_max_, Ω s^−2^), and systolic flow time (T_SF_, s) from fiducial landmarks on the dZ/dt waveform [15, 20] (Fig. 2a, b). Finally, to obtain ohmic mean velocity, the quotient of dZ/dt_max_ and Z_0_ undergo square root transformation. To obtain SV, a volumetric constant (V_C_, mL) is calculated from total body weight (W, kg), whereupon the variables are placed into the SV equation (vide infra).

The TBEV analog electronics were identical across all subjects. The waveforms for subjects 1 through 13 were digitized in real-time using a BIOPAC UIM100C system (BIOPAC, Goleta, CA, USA) and then stored on a laptop PC for later analysis. All waveforms were sampled at 16-bit resolution and 500 Hz. In addition, A BIOPAC ECG100C module was used to collect a single ECG lead II waveform for determining HR and gating of dZ/dt. Subjects 14 though 32 were digitized using an integrated digital sub-system on the TBEV circuit board (Sotera Wireless, San Diego, CA, 92121, USA). With regard to the two prototype impedance platforms, they were operationally identical for signal acquisition and data processing.

#### Assumptions of the method [15, 20, 21]

It is assumed that;

1. Schematically, the brachium can be considered a thin-walled cylinder, encasing an aggregate of adynamic tissues of high impedance, which, in turn, encircle a single dynamic blood-filled vascular conduit of low impedance.
2. When an ac field is applied longitudinally over a segment of the brachium, the brachium can be considered, bio-electrically, a parallel conduction curcuit, connecting (1) a poorly conductive static tissue impedance, Z_t_, (2) a highly conductive blood resistance (R_b_, Z_b_), and (3) the smallest volume, but highest conductivity impedance, reflecting the quasi-static interstitial tissue water, (Z_w_), varying with tissue hydration. The parallel (║) reciprocal sum, Z_t_^−1^ + Z_b_^−1^ + Z_w_^−1^, constitutes the quasi-static base impedance, Z_0_. When the static and quasi-static tissue impedances are added in parallel with the time-dependent, dynamic pulsatile component of the blood resistance (∆R_b_(t) ≡ ∆Z_b_(t)), the total transbrachial impedance, Z(t) results. As per Ohm’s Law (vide supra); when an ac field is uniformly applied longitudinally across both the static and dynamic impedances, a potential difference between the current injecting electrodes produces the parallel connection of a static and dynamic voltage; U_0_^−1^ and ∆U_b_(t)^−1^, respectively. In concise bio-electric mathematical language,
  1 $$I(t) \cdot \left[ {\left( {\left\| {Z_{t} } \right\|Z_{b} \left\| {Z_{w} } \right.} \right)\left\| {\Delta Z_{b} (t)} \right.} \right] = \left. {U_{0} } \right\|\Delta U_{b} (t)$$
  A schematic electronic circuit diagram reflecting Eq. 1 is given as follows (Fig. 3).

**Fig. 3:**
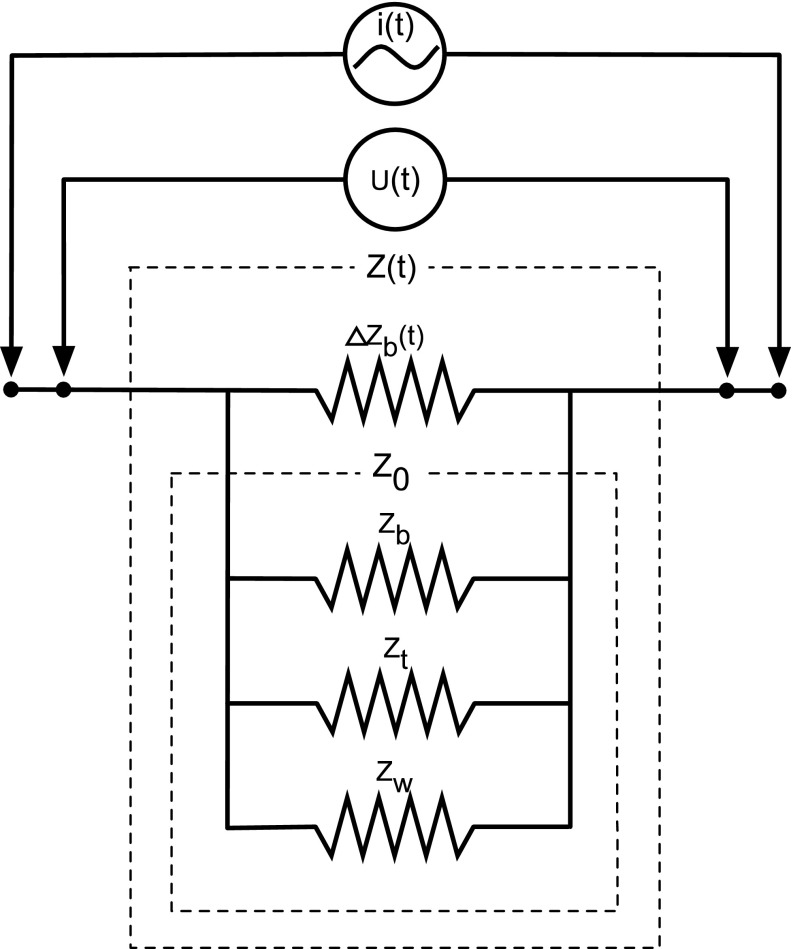
The transbrachial impedance (Z(t)) comprises four elements. The three static elements include the impedance path through the tissue (Z_t_), the blood (Z_b_), and the interstitial water (Z_w_). The parallel connection of these static impedances constitutes the transbrachial base impedance, Z_0_. The parallel connection between Z_0_ and the pulsatile component, ∆Z_b_(t), constitutes the total impedance of the brachium (Z(t)). Shown is the current input (I(t)) and time variable voltage output (U(t))
3. The brachial artery is poorly compliant and mechanically resembles a rigid muscular tube, rendering volume changes trivial. Thus,
4. All cardiogenically-induced impedance changes (∆Z_b_(t)) are due solely to blood velocity-induced resistivity changes (∆ρ_b_(t), Ω cm s^−1^)
5. The velocity-induced change in blood resistivity is due to the changing orientation of the bi-concave, disc-shaped erythrocytes from random orientation in end-diastole (state of lowest conductivity) to parallel orientation along their long axis of symmetry (state of highest conductivity) in the rapid ejection phase of systole
6. Transbrachial ∆Z_b_(t) (Ω s^−1^) is an ohmic analog of brachial artery blood velocity (cm s^−1^), which implies that its peak first time-derivative, dZ/dt_max_ (Ω s^−2^), is an acceleration analog
7. Transbrachial dZ/dt_max_ (Ω s^−2^) peaks precisely with brachial artery peak blood acceleration (cm s^−2^)
8. Peak brachial artery acceleration (cm s^−2^) is directly proportional to the magnitude and change of magnitude of peak aortic blood acceleration (cm s^−2^)
9. Peak transthoracic dZ/dt (dZ/dt_max_) is directly proportional to the magnitude and change in magnitude of peak aortic blood acceleration
10. Transbrachial dZ/dt_max_ is directly proportional to the magnitude and change in magnitude of transthoracic dZ/dt_max_ and thus,
11. Transbrachial dZ/dt_max_ is directly proportional to the magnitude and changes in magnitude of peak aortic blood acceleration.
12. To obtain dimensionless ohmic mean velocity (s^−1^), square root transformation of dZ/dt_maz_/Z_0_ is necessary
13. A constant ratio of 2:1 exists between the magnitudes of transthoracic ohmic mean velocity and transbrachial ohmic mean velocity, respectively. Thus, reciprocally,
14. A constant ratio of 1:2 exists between the magnitudes of the transthoracic and transbrachial V_C_, respectively
15. The magnitude of the transthoracic constant of proportionality, otherwise known as the volume conductor (V_C_, mL) is equivalent to the global end-diastolic volume (GEDV, mL)

#### TBEV data collection protocol

Two 5-min sessions of TBEV waveforms were collected. The first was collected immediately prior to the cMRI, and the second immediately following the cMRI. The subjects remained supine from the start of the first measurement, through the cMRI, and until the end of the second TBEV measurement. The subjects were instructed to remain silent and still for the duration of the measurements.

#### TBEV signal processing

First, a 30 s interval out of each 5 min period was selected for analysis. By default, this period was 150–180 s, but if any significant artifact was present upon operator inspection, then a different 30-s period was selected manually. To find a mean value over the relevant time interval, signal averaging was implemented. This was the only manual intervention in the analysis. TBEV SV was then calculated according to the equation of Bernstein et al. [15].

2 $$SV_{TBEV} = V_{C} \cdot \sqrt {\frac{{dZ/dt_{\hbox{max} } }}{{Z_{0} }}} \cdot T_{SF}$$

- V_C_ = volume conductor (mL) = 32 × W^1.02^, where W = total body weight (kg)
- dZ/dt_max_ = peak time rate of change of the transbrachial impedance pulse variation (Ω s^−2^)
- Z_0_ = quasi-static transbrachial base impedance (Ω)
- T_SF_ = systolic flow time (s) ≡ left ventricular ejection time (s)
- $\sqrt {\frac{{dZ/dt_{\hbox{max} } }}{{Z_{0} }}}$ = ohmic mean velocity (s^−1^)

Rationale for a mass-based V_C_, the square root transformation, and the unit assignment of Ω s^−2^ for dZ/dt_max_ are discussed extensively elsewhere [15, 20, 21].

#### Statistical analysis

For each group of subjects, to assess reproducibility and accuracy, we employed Bland–Altman method and calculated mean bias, standard deviations (SD), precision, limits of agreement (LOA), and percent error for absolute values of the continuous parameters studied [22]. Pearson’s correlation coefficient (r^2^) was calculated and used to assess the closeness of association between continuous variables. For statistical analysis we used R version 3.0.0 (2013-04-03), Copyright (C) 2013, The R Foundation for Statistical Computing.

## Results

### Demographics and echocardiography

The study population demographics are summarized in Table 1. There were 24 males, the mean age was 41.5 ± 12.5 years, and the mean body mass index (BMI) was 26 ± 4.4 kg m^−2^ and ranged from 19 to 35 kg m^−2^. All subjects were in normal sinus rhythm. Upon Doppler echocardiographic interrogation, all subjects had trace or no mitral regurgitation. One subject had mild aortic regurgitation; all other subjects had trace or no aortic regurgitation.

### cMRI

High quality cMRI images were obtained in all subjects (n = 32) and displayed well-defined endocardial borders that allowed calculation of left ventricular (LV) SV and CO. Values for these and other cMRI parameters are listed in Table 1. The average ejection fraction was 62.2 ± 4.6 %, and the average CO was 6.08 ± 1.3 L min^−1^, with a mean cardiac index of 3.00 ± 0.41 L min^−1^ m^−2^.

### TBEV

The TBEV units functioned as intended, with high quality waveform and digital data obtained in all but three subjects. In two subjects, who were evaluated the same day, data acquisition equipment malfunction occurred, and hence no useable TBEV data was obtained. In a third subject, the pre-MRI TBEV data was accidentally over-written due to operator error and therefore was unavailable. Hence, full datasets were obtained in 29 subjects, and for a 30th subject, only the post-MRI TBEV data was obtained. In a fourth subject, the disparity between pre- and post-cMRI SV was 33 mL (88.73–55.78 mL), which translates to Bland–Altman percent difference of 45.7 %. The corresponding cMRI SV was 102.2 mL. This disparity was probably due to a spurious value of Z_0_ for the post TBEV measurement. Pre-TBEV Z_0_ was 60 versus 100 Ω for the post MRI Z_0_. This is impossible, because there was no change in brachial tissue extra-cellular water over the cMRI interval. The disparity, no doubt, was due to a defective electrode or faulty adhesion of the electrode-to-skin interface. Despite this obvious outlier, the paired estimates were included in statistical analysis. Raw TBEV input and output variables are shown in Table 2. The results demonstrate that, for pre- and post-cMRI, the mean values for T_SF_, Z_0_, SV, HR and CO are virtually unchanged for males and females. The largest mean difference from pre- to post-cMRI occurred in dZ/dt_max_ for both males (9 %) and females (6 %). Also of interest, the mean value of the magnitude of the male V_C_ is approximately 50 % larger than the female counterpart. If the mean value of the magnitude of ohmic mean velocity is calculated from the data (see Eq. 2), female values are about 18 % higher than the male cohort. In conjunction with a 3 % longer T_FS_ in females, the difference between males and females renders SV in males approximately 25 to 30 % higher. A total of 30 subjects were used in statistical analysis (n = 30).

**Table 2:** TBEV equation input and output variables: V_C_ = volume conductor (mL), T_SF_ = systolic flow time (ms), dZ/dt_max_ = peak time rate of change of the transbrachial impedance pulse variation (Ω s^−2^, ohm s^−2^), Z_0_ = transbrachial quasi-static base impedance (Ω), SV = stroke volume (mL), HR = heart rate (beats min^−1^), CO = cardiac output (L min^−1^)

Pre-MRI session	Males (n = 21)		Females (n = 8)	
	Mean ± SD	Range	Mean ± SD	Range
V_c_ (mL)	2929 ± 458	2144–4000	1914 ± 541	1571–3286
T_SF_ (ms)	321 ± 19.2	276–356	328.8 ± 25.3	296–388
dZ/dt_max_ (Ω s^−2^)	0.97 ± 0.39	0.47–1.92	1.60 ± 0.41	1.03–2.46
Z_0_ (Ω)	74.3 ± 11	49–96	86.6 ± 15.4	64.9–113.8
SV (mL)	104.1 ± 16.3	75.9–143.8	83.8 ± 19.9	70.7–135.3
HR (BPM)	67 ± 8	53–86	61 ± 7	55–71
CO (L min^−1^)	6.9 ± 1.1	5.1–9.4	5.2 ± 1.7	3.9–9.7

Post-MRI session	Males (n = 22)		Females (n = 8)	
	Mean ± SD	Range	Mean ± SD	Range
V_c_ (mL)	2935 ± 448	2144–4000	1914 ± 541	1571–3286
T_SF_ (ms)	318 ± 22.7	278–372	328 ± 32.3	288–384
dZ/dt_max_ (Ω s^−2^)	0.89 ± 0.29	0.42–1.67	1.51 ± 0.52	0.86–2.65
Z_0_ (Ω)	72.1 ± 14.2	41.8–100.0	90.0 ± 20.7	51.4–117.8
SV (mL)	102.5 ± 18.4	55.8–138.2	80.0 ± 20.4	54.4–128.3
HR (BPM)	67 ± 8	57–86	61 ± 7	50–72
CO (L min^−1^)	6.8 ± 1.3	3.4–8.7	5.0 ± 1.7	3.6–8.9

### Bland–Altman: mean bias, precision, and limits of agreement (LOA)

**Fig. 4:**
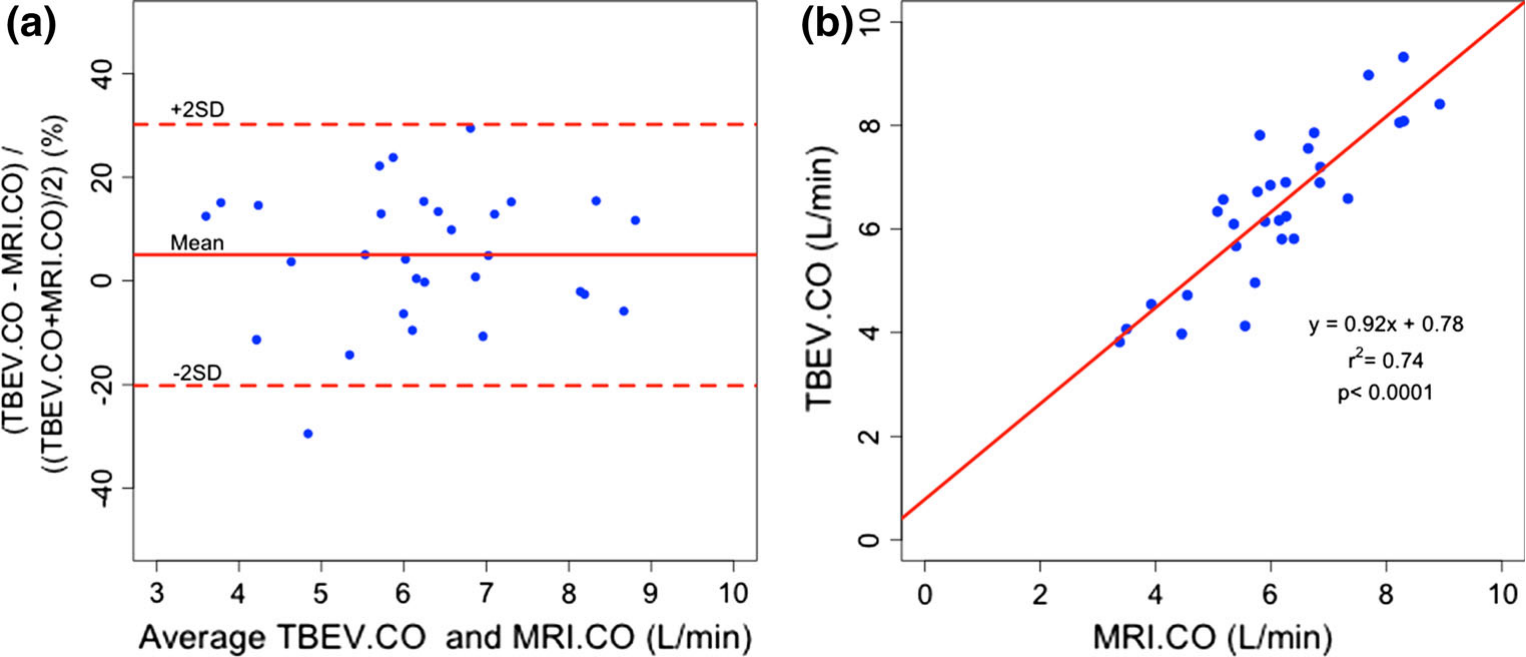
**a** Bland–Altman analysis: average TBEV CO versus cMRI CO See Table 3 for mean bias precision, ±LOA, and percent error. **b** Correlation analysis for TBEV CO versus cMRI CO: the slope (0.92) and y-intercept (0.78) suggest little systematic bias. See Table 4

Mean bias, precision, mean bias ± 1.96 × SD (LOA), and percent error for TBEV SV versus cMRI SV and TBEV CO versus cMRI CO are shown in Table 3. Of note in Table 3 are the small biases, similar levels of precision, and LOA for the three paired comparisons. Percent error for TBEV SV versus cMRI SV and TBEV CO versus cMRI CO are virtually identical, being 26.2 and 24.8 %, respectively. Since cMRI SV is essentially without precision error, the disparity in measurements of SV and CO between the methods can be attributed almost entirely to the precision errors of TBEV. Bland–Altman methodology was used to assess comparisons of TBEV pre- versus post-cMRI SV, averaged TBEV SV versus cMRI SV and averaged TBEV CO versus cMRI CO. This analysis also assumes that true SV remained unchanged over the cMRI interval. Table 3 and Fig. 4a show results of Bland–Altman analysis for TBEV CO versus cMRI CO.

**Table 3:** Bland–Altman analysis: the aggregate mean value of the paired values are presented as mean bias, precision, and ±95 % limits of agreement (LOA), which are presented as percent (%) and mL

	Mean bias (%)	Precision (%)	95 % Limits of agreement		
			Upper (%)	Lower (%)	% Error
TBEV SV-1 versus TBEV SV-2	2.87	13.59	+29.51	−23.77	–
TBEV SV versus cMRI SV	−1.56	13.47	+24.85	−27.97	–
TBEV CO versus cMRI CO	5.01	12.85	+30.20	−20.17	–

	Mean bias (mL)	Precision (mL)	95 % Limits of agreement		
			Upper (mL)	Lower (mL)	% Error
TBEV SV-1 versus TBEV SV-2	2.05	11.99	+25.55	−21.45	–
TBEV SV versus cMRI SV	−1.53	12.84	+23.64	−26.71	26.2

	Mean bias (L min^−1^)	Precision (L min^−1^)	Upper (L min^−1^)	Lower (L min^−1^)	
TBEV CO versus cMRI CO	0.32	0.77	+1.83	−1.19	24.8

Percent error is computed for absolute values of SV and CO. For cardiac output (CO) mean bias, precision, and ±95 % LOA are presented as (%) and L min^−1^. Note that the mean biases are small and the SD, precision and limits of agreement are similar over the 3 comparisons

### Linear regression analysis

The regression coefficients, y-intercepts, and correlation coefficients for three paired comparisons are presented in Table 4. It is interesting to note that, for TBEV SV versus cMRI SV is the lowest of the three comparisons with a regression coefficient of 0.82 and correlation coefficient of r^2^ = 0.61 (r = 0.78). The regression coefficient in Fig. 4b is a scatterplot of TBEV CO versus cMRI CO, showing a correlation coefficient r^2^ = 0.74 (r = 0.86), a regression coefficient close to unity (i.e. 0.92), and a y-intercept less than 1 (one) L min^−1^ (i.e. 0.78 L min^−1^). This suggests little systematic bias of CO between the methods. Due to the absolute precision of cMRI SV, the scatter around the regression line represents random errors of the TBEV method.

**Table 4:** Linear regression analysis for TBEV SV-1 (pre-) versus TBEV SV-2 (post), Average TBEV SV versus cMRI SV, and average TBEV CO versus cMRI CO

TBEV and cMRI comparisons	Regression equation (y = mx + b)	Correlation coefficient (r^2^, r)
TBEV SV-1 versus TBEV SV-2	y = 0.76x + 25.1	r^2^ = 0.71, r = 0.84
Average TBEV SV versus cMRI SV	y = 0.82x + 19.1	r^2^ = 0.61, r = 0.78
Average TBEV CO versus cMRI CO	y = 0.92x + 0.78	r^2^ = 0.74, r = 0.86

Note that for CO the slope is nearly 1 and the y-intercept <1 L min^−1^

## Discussion

### Recent studies and historical aspects

Results obtained in this study are similar to our previous study in normals, comparing TBEV SV to Doppler/echo-derived SV [15, 20]. By Bland–Altman method, the mean bias was −4.0 % (2.5 mL), precision of 14.7 % (11.9 mL), and limits of agreement of +33.4 and −25.4 %. Contrary to the sequence of method comparisons in this study, which were spaced-apart temporally, in our prior study Doppler/echo and TBEV SV measurements were obtained simultaneously.

Transbrachial SV determination is not without historical precedent. It is interesting to note that, in Nyboer’s seminal article in 1950, he used a 4 band-electrode montage on the forearm to obtain plethysmographic (volumetric) impedance measurements [23]. That article also presented the original equations that evolved into those used in transthoracic ICG [21]. Using a similar electrode array to ours, a more recent study, assessing cardiogenically-induced bioimpedance changes of the forearm, has shown close correspondence between SV changes and percent changes obtained from the aortic root and the changes and percent changes in both the maximum amplitude of the impedance pulse variation ∆Z_max_ (r = 0.86 and r = 0.84, respectively) and the area beneath the ∆Z waveform envelope (r = 0.78 and r = 0.78, respectively) [24].

### Strength and advantages of TBEV

First, in contrast to the transthoracic approach, there are virtually no potentially confounding or competing vessel resistivities or volume changes in the upper arm. The sole vessel of any magnitude is the brachial artery, which is probably the only major source providing a waveform for signal processing. Unlike transthoracic dZ/dt, which has multiple signal sources and both a volumetric and blood velocity-induced resistivity component [25], the brachial artery is sufficiently stiff to yield a signal whose origin is purely reflective of axial blood flow [15]. Secondly, unlike the transthoracic method, which is highly susceptible to excess extravascular lung water [13, 14], brachial measurements are taken distant from the lung and apart from the bony thorax. As a consequence of the upper arm being detached from the thorax, the wide respiratory-induced oscillations of baseline impedance (Z_0_), seen in the transthoracic approach, are much attenuated in the upper arm. As concerns signal processing, this improves detection of baseline impedance Z_0_, leading to fewer morphologic distortions of ∆Z_b_(t), thus resulting in less need for adaptive filtering.

### Limitations and errors of the method and study design

The results from this study suggest that the theory and equations are, at least in the first approximation, correct. However, it must be remembered that this study was conducted with healthy volunteers who were apparently free of heart disease. Potential mechanical limitations of the method entail atherosclerotic obstructive disease of the subclavian and axillo-brachial circulations, namely subclavian and axillo-brachial stenosis [26]. Stenosis induces high velocity jets, blood turbulence, and disturbed laminar flow, precluding accurate SV determination [27]. Hemodynamically, abnormal ejection patterns can potentially cause distortion of the brachial artery waveform envelope, thereby preventing accurate signal processing [27, 28]. Clearly, random errors in measurement of dZ/dt_max_, Z_0_, and systolic flow time (T_SF_) can degrade the accuracy of the method. Operationally, defective electrodes will yield invalid results.

Two serious design flaws in our study protocol entailed; (1) the non-simultaneity of the TBEV and cMRI measurements and (2) the lack of a gold-standard continuous method by which to assess TBEV’s accuracy and tracking/trending capability over a wide range of hemodynamic perturbations. In this regard, we were unable to completely verify assumptions 8 through 11 (Sect. 2.5.2). Concerning lack of simultaneity of the TBEV measurements, the following is relevant; since all metallic objects of ferric origin are contraindicated while undergoing cMRI, the TBEV cables, electrodes, and wrist-worn device were obligatorily disconnected and then reconnected. It was originally assumed that TBEV SV would not change during the cMRI interval, and if it did, only minimally. Judging by the precision and LOA of the two TBEV comparison measurements (Table 3), it is difficult to determine whether SV actually changed, or was simply a function of the imprecision of the method. Since the two TBEV measurements were separated by about an hour, a combination of precision error and actual change in SV probably occurred. It is thus speculated that, had the comparison between simultaneous measurements of TBEV SV versus cMRI SV been technically possible, the precision and accuracy may have been appreciably better. Again, regarding the non-simultaneity of TBEV versus cMRI measurements, it was our objective to compare precision and accuracy with an absolute measurement of SV. Clearly, it was an erroneous assumption that the two TBEV measurements would remain constant over the cMRI interval, or that the average of the two measurements would represent the time-averaged cMRI SV over the scanning period.

As concerns clinical efficacy, it is not known how changes in loading conditions or administration of vasodilators, vasoconstrictors or inotropic medications will affect the accuracy of the TBEV measurement. On this very issue, Chemla et al. [29, 31] found that, unlike peak velocity, brachial artery peak acceleration was unaffected by downstream vasoactivity and only by positive or negative β_1_ inotropic and chronotropic medications directly affecting the adrenoceptors of the myocardium. This suggests that ohmic peak brachial artery acceleration (i.e. dZ/dt_max_) may respond similarly.

Other limitations of the study include a cohort mainly of males (22/8) and the inability to obtain TBEV waveforms in the presence of arm or body motion or complex dysrhythmias, including atrial fibrillation. While the implementation of two prototype impedance platforms might be perceived as a limitation, they were operationally identical for signal acquisition and data processing.

### Assumptions and inherent errors of the method

#### A constant ratio of 2:1 exists between the magnitudes of transthoracic and transbrachial ohmic mean velocity

A basic assumption of the method assumes that using transthoracic dZ/dt_max_ and the parent equation of Eq. 1 [30], results in valid approximations of SV. As described in prior publications, the accuracy and precision of TBEV SV depends upon a monotonically fixed ratio between the magnitudes and changes in magnitude of peak aortic and peak brachial artery blood acceleration (d*v*/dt_max_, cm s^−2^; dQ/dt_max_, mL s^−2^) [15, 20, 31]. Since transthoracic and transbrachial dZ/dt_max_ are considered ohmic analogs of peak aortic and peak brachial artery blood acceleration, respectively [15, 31], it was hypothesized that SV could be obtained from the brachial artery using the skeleton of Eq. 1 (vide supra) [30]. Pursuant to that hypothesis, it was found that the ratio of transthoracic to transbrachial ohmic mean velocity ([dZ/dt_max_·Z_0_^−1^]^0.5^, s^−1^) was approximately 2 to 1 (i.e. 2:1) [15, 20]. Comparing the latter to the former, we found a mean bias of −46.4 % (~50 %), a transthoracic to transbrachial mean ratio of 2.16:1, and percent deviation from 2:1 of 8 %. In 36 of the 38 study subjects (94.7 %), paired data points were equal to or less than 15 % from the mean [20]. Thus, at the operational level, and as an inherent pre-requisite assumption of the method, the direct proportionality and a constant 2:1 ratio between transthoracic and transbrachial ohmic mean velocity may not exist in an unknown patient population. These ratios may even change in the same individual over time and may account for some portion of the precision error between pre- and post-cMRI TBEV SV measurements (Table 3). This can lead to an unpredictable degree of uncontrollable under or overestimation of transbrachial ohmic mean velocity, and thus SV. Thus, assumption 13 (Sect. 2.5.2) is only an approximation.

#### An allometric relationship between body weight and GEDV is a constant

Since the ratio of transthoracic to transbrachial ohmic mean velocity is approximately 2:1, and in an abstraction of the equation of continuity for conservation of mass flow, the ratio of the magnitude of transthoracic to the transbrachial V_C_ was set empirically and reciprocally at 1:2. Theoretically, the physical embodiment of the transthoracic V_C_ was estimated to approximate the GEDV. Thus, the allometric relationship of body weight to GEDV was empirically approximated as 16W^1.02^ (~17 mL kg^−1^) [30].^1^ For allometrics, the relationship of body weight to GEDV is given as aM^b^, where “a” is a constant of proportionality, M is patient body mass (kg), and “b” is an exponential constant describing linearity or non-linearity [32]. The exponential constant, 1.02, implies a linear relationship between body weight and GEDV. Conversion of body weight directly to blood volume is valid, because the density of blood (ρ, g mL^−1^) is 1.05–1.06. When empirically derived GEDV is multiplied by a mean value of 1.25, the generally accepted mean correction factor for conversion to intrathoracic blood volume (ITBV) (range 1.21–1.45) [33], 21.25 mL kg^−1^ is the result. This empirically-derived calculated value was verified experimentally by Moller et al. [34] as the indexed mean ITBV for patients without cirrhosis. Further interpretation of their data for these patients yielded a pooled SD of approximately ±4.7 mL kg^−1^, corresponding to ±22 %. By mathematical coupling, this percent error is fully transferrable to the GEDV. Corroborating the aforementioned studies, data provided by Metzelder et al. [35] show that, for a mean control ITBV of 1638 mL and a mean weight for patients of 74 kg, the mean calculated indexed ITBV was 22.14 mL kg^−1^. When this value is divided by 1.25 to obtain GEDV, the result is 17.7 mL kg^−1^. This value is 4.7 % greater than using the transthoracic coefficient and exponent, 16^1.02^ (i.e. 16.9 mL kg^−1^). While the measured mean indexed values for the ITBV are well described by the allometric expression, 16W^1.02^ multiplied by 1.25 [33], the rather substantial standard deviations, especially those reported by Moller et al. [34], contribute to the error of the fixed-value transbrachial coefficient and exponent, 32^1.02^ mL kg^−1^. As is true for assumption 13 (Sect. 2.5.2), assumption 15 is an approximation.

#### Transbrachial dZ/dt_max_ is an ohmic analog of peak brachial artery acceleration

Transthoracically, the assumption that dZ/dt_max_ peaks synchronously with the I wave of the acceleration ballistocardiogram (aBCG) [21, 30], in the time-domain, has recently been challenged. Luchitskaya et al. and Ulbrich et al. have shown experimentally that, dZ/dt_max_ peaks ~23–28 ms after peak acceleration of blood flow [36, 37], but before peak aortic blood flow (dV/dt_max_). This finding would appear to somewhat question the acceleratory nature of transthoracic dZ/dt_max_ and necessity for the square root acceleration step-down transformation to obtain ohmic mean velocity (Sect. 2.5.2 assumptions 7–12). Operationally, however, when implementing the transthoracic technique, the parent equation of Eq. 2 has been validated extensively according to assumption 12 (Sect. 2.5.2) [38–43]. Experimentally, it has been definitively demonstrated that an equation using square root transformation of dZ/dt_max_/Z_0_ provides better agreement and correlation with TDCO than equations which assume a volumetric origin for dZ/dt_max_ [30]. Despite the findings disputing the peak acceleratory origin of transthoracic dZ/dt_max_ [36, 37], pictorial and analytical evidence provided by Bernstein [21] clearly indicates that dZ/dt_max_ peaks synchronously with peak aortic blood acceleration and I wave of the aBCG. Thus, although transthoracic dZ/dt_max_ may not peak precisely with peak acceleration, but closely within the time domain, it can be treated as such. Moreover, with respect to the transbrachial method, it has been definitively demonstrated that peak Doppler brachial artery blood acceleration and peak brachial artery ohmic acceleration reach their magnitudes synchronously in the time domain [15, figure 8c, d]. This appears to validate assumption 7 (Sect. 2.5.2).

It should be fully appreciated that the TBEV technique may not provide credible SV or CO values in the presence of critical illness. However, this and our previous study [15], both showing proof of concept, serve as evidence that SV can be obtained by TBEV technique in the ideal homeostatic hemodynamic state. Future validation studies in sick humans, comparing TBEV to another validated continuous SV/CO technique, such as in the studies by Metzelder et al. [35] and Lorne et al. [44], will determine the viability of TBEV in clinical medicine.

## Conclusion

In this study, we present a comparison of SV and CO obtained via electrical interrogation of the brachial artery by the TBEV technique with SV and CO obtained by cMRI in normals. Despite its potential sources of error, we found that TBEV resulted in satisfactory reproducibility and agreement with cMRI. While not interchangeable with cMRI, based on existing criteria for the reproducibility of pulmonary artery TDCO [45, 46] and its LOA, [46] the precision and accuracy of TBEV CO obtained in this study may be clinically acceptable if maintained in the critically ill.

## Footnotes

1 In earlier publications [15, 23, 31], the V_C_ was erroneously thought to approximate the ITBV.

## References

[1] Pinsky MR (2007). Hemodynamic evaluation and monitoring in the ICU. Chest.

[2] Reuter DA, Huang C, Edrich T, Sherman SK, Eltzschig HK (2010). Cardiac output monitoring using indicator dilution techniques: basics, limitations, and perspectives. Anesth Analg.

[3] Lee AJ, Cohn JH, Ranasinghe JS (2011). Cardiac output assessed by invasive and minimally invasive techniques. Anesthesiol Res Pract.

[4] Della Rocca G, Costa GM, Coccia C, Pompei L, Di Marco P, Pietropaoli P (2002). Preload index: pulmonary artery occlusion pressure versus intrathoracic blood volume monitoring during lung transplantation. Anesth Analg.

[5] Kumar A, Anel R, Bunnell E, Habet K, Zanotti S, Marshall S, Neumann A, Ali A, Cheang M, Kavinsky C, Parrillo JE (2004). Pulmonary artery occlusion pressure and central venous pressure fail to predict ventricular filling volume, cardiac performance, or the response to volume infusion in normal subjects. Crit Care Med.

[6] Chatterjee K (2009). The Swan-Ganz catheters: past, present, and future: a viewpoint. Circulation.

[7] Marik PE (2013). Obituary: pulmonary artery catheter 1970 to 2013. Ann Intensive Care.

[8] Peyton PJ, Chong SW (2010). Minimally invasive measurement of cardiac output during surgery and critical care: a meta-analysis of accuracy and precision. Anesthesiology.

[9] Truijen J, van Lieshout JJ, Wesselink WA, Westerhof BE (2012). Noninvasive continuous hemodynamic monitoring. J Clin Monit Comput.

[10] Marik PE (2013). Noninvasive cardiac output monitors: a state-of-the-art review. J Cardiothorac Vasc Anesth.

[11] Kamath SA, Drazner MH, Tasissa G, Rogers JG, Stevenson LM, Yancy CW (2009). Correlation of impedance cardiography with invasive hemodynamic measurements in patients with advanced heart failure: bioimpedance cardiography (BIG) substudy of the ESCAPE Trial. Am Heart J.

[12] Raaijmakers E, Faes TJ, Scholten RJ, Goovaerts HG, Heethaar RM (1999). A meta-analysis of three decades of validating thoracic impedance cardiography. Crit Care Med.

[13] Peng ZY, Critchley LA, Fok BS (2005). An investigation to show the effect of lung fluid on impedance cardiac output in the anesthetized dog. Br J Anaesth.

[14] Critchley LA, Calcroft RM, Tan PY, Kew J, Critchley JA (2000). The effect of lung injury and excessive lung fluid, on impedance cardiac output measurements, in the critically ill. Intensive Care Med.

[15] Bernstein DP, Henry IC, Banet MJ, Dittrich T (2012). Stroke volume obtained by electrical interrogation of the brachial artery: transbrachial electrical bioimpedance velocimetry. Physiol Meas.

[16] LaGerche A, Claessen G, Van de Bruaene A, Pattyn N, Van Cleemput J, Gewillig M, Bogaert J, Dymarkowski S, Claus P, Heidbuchel H (2013). Cardiac MRI: a new gold standard for ventricular volume quantification during high-intensity exercise. Circ Cardiovasc Imaging.

[17] Pennell DJ (2010). Cardiovascular magnetic resonance. Circulation.

[18] Mosteller RD (1987). Simplified calculation of body-surface area. N Engl J Med.

[19] Quinones MA, Otto CM, Stoddard M, Waggoner A, Zoghbi WA (2002). Recommendations for quantification of Doppler echocardiography. a report from the Doppler quantification task force of the nomenclature and standards committee of the American Society of Echocardiography. J Am Soc Echocardiogr.

[20] Henry IC, Bernstein DP, Banet MJ (2012). Stroke volume obtained from the brachial artery using transbrachial bioimpedance velocimetry. Conf Proc IEEE Eng Med Biol Soc.

[21] Bernstein DP (2010). Impedance cardiography: pulsatile blood flow and the biophysical and electrodynamic basis for the stroke volume equations. J Electr Bioimp.

[22] Bland JM, Altman DG (1986). Statistical methods for assessing agreement between two methods of clinical measurement. Lancet.

[23] Nyboer J (1950). Electrical impedance plethysmography. A physical and physiological approach to peripheral vascular study. Circulation.

[24] Wang JJ, Wang PW, Liu CP, Lin SK, Hu WC, Dao T. Evaluation of changes in cardiac output from the electrical impedance of the forearm. Physiol Meas. 2007;28:989–99. 10.1088/0967-3334/28/9/002 17827648

[25] Wang L, Patterson R. Multiple sources of the impedance cardiogram based on 3-D finite difference human thorax models. IEEE Trans Biomed Eng. 1995;42:141–48. 10.1109/10.341826 7868141

[26] Ochoa VM, Yeghiazarians Y (2010). Subclavian artery stenosis: a review for the vascular medicine practitioner. Vasc Med.

[27] Gault JH, Ross J, Mason DT (1966). Patterns of brachial arterial blood flow in conscious human subjects with and without cardiac dysfunction. Circulation.

[28] Guler N, Bilge M, Eryonucu B, Erkoc R, Ipeksoy U (2000). Brachial artery blood flow velocity pattern in patients with congestive heart failure: duplex Doppler ultrasonographic assessment. Angiology.

[29] Chemla D, Levenson J, Valensi P, LeCarpentier Y, Pourny JC, Pithois-Merli I, Simon A (1990). Effect of beta adrenoceptors and thyroid hormones on velocity and acceleration of peripheral arterial flow in hyperthyroidism. Am J Cardiol.

[30] Bernstein DP, Lemmens HJ (2005). Stroke volume equation for impedance cardiography. Med Biol Eng Comput.

[31] Chemla D, Demolis P, Thyrault M, Annane D, Lecarpentier Y, Giudicelli JF (1996). Blood flow acceleration in the carotid and brachial arteries: respective contributions of cardiac performance and local resistance. Fundam Clin Pharmacol.

[32] Lindstedt SL, Schaeffer PJ (2002). Use of allometry in predicting anatomical and physiological parameters of mammals. Lab Anim.

[33] Nirmalan M, Willard TM, Edwards DJ, Little RA, Dark PM (2005). Estimation of errors in determining intrathoracic blood volume using single transpulmonary thermodilution technique in hypovolemic shock. Anesthesiology.

[34] Moller S, Henriksen JH, Bendtsen F (2003). Central and noncentral blood volumes in cirrhosis: relationship to anthropometrics and gender. Am J Physiol Gastrointest Liver Physiol.

[35] Metzelder SM, Coburn M, Stoppe C, Fries M, Simon T-P, Reinges MH, Hollig A, Rossaint R, Marx G, Rex S (2014). Accuracy and precision of calibrated arterial pulse contour analysis in patients with subarachnoid hemorrhage requiring high-dose vasopressor therapy: a prospective observational clinical trial. Crit Care.

[36] Luchitskaya E, Deliere A, Diedrich N, Almorad A, Beck P, Gauger P, Limper U, Funtova I, Baevsky RM, Migeotte PF, Tank J (2013). Timing and source of the maximum of the transthoracic impedance cardiogram (dZ/dt) in relation to the H-I-J complex of the longitudinal ballistocardiogram under gravity and microgravity conditions. Conf Proc IEEE Eng Med Biol Soc..

[37] Ulbrich M, Muhlsteff J, Leonhardt S, Walter M. Influence of physiologic sources on the impedance cardiogram analyzed using 4D FEM simulations. Physiol Meas. 2014;35:1451–68. 10.1088/0967-3334/35/7/1451 24901446

[38] Norozi K, Beck C, Osthaus WA, Wille I, Wessel A, Bertram H (2008). Electrical velocimetry for measuring cardiac output in children with congenital heart disease. Br J Anaesth.

[39] Zoremba N, Bickenbach IJ, Krauss B, Rossaint R, Kuhlen R, Schalte G (2007). Comparison of electrical velocimetry and thermodilution techniques for the measurement of cardiac output. Acta Anaesthesiol Scand.

[40] Noori S, Drabu B, Soleymani S, Seri I (2012) Continuous non-invasive cardiac output measurements in the neonate by electrical velocimetry: a comparison with echocardiography. Arch Dis Child Fetal Neonate Ed 97(5):F340–3. doi:10.1136/fetalneonatal-2011-301090. 10.1136/fetalneonatal-2011-301090 22933092

[41] Rauch R, Welisch E, Lansdell N, Burrill E, Jones J, Robinson T, Bock D, Clarson C, Filler G, Norozi K (2012) Non-invasive measurement of cardiac output in obese children and adolescents: comparison of electrical cardiometry and transthoracic Doppler echocardiography. J Clin Monit Comput 27(2):187–93. doi:10.1007/s10877-012-9412-7 [Epub 2012 Nov 21]. 10.1007/s10877-012-9412-7 23179019

[42] Grollmuss O, Gonzalez P (2014). Non-invasive cardiac output measurement in low and very low birth weight infants: a method comparison. Front Pediatr.

[43] Grollmuss O, Demontoux S, Capderou A, Serraf A, Belli E (2012) Electrical velocimetry as a tool for measuring cardiac output in small infants after heart surgery. Intensive Care Med 38(6):1032–9. doi:10.1007/s00134-012-2530-3 [Epub 2012 Mar 30]. 10.1007/s00134-012-2530-3 22460851

[44] Lorne E, Mahjoub Y, Diouf M, Sleghem J, Buchalet C, Guinot PG, Petiot S, Kessavane A, Dehedin B, Dupont H (2014) Accuracy of impedance cardiography for evaluating trend in cardiac output: a comparison with oesophageal Doppler. Br J Anaesth 28. Pii: aeu136 [Epub ahead of print]. 10.1093/bja/aeu136 24871872

[45] Critchley LA, Critchley JA (1999). A meta-analysis of studies using bias and precision statistics to compare cardiac output techniques. J Clin Monit Comput.

[46] Cecconi M, Rhodes A, Poloniecki J, Della Rocca G, Grounds RM (2009). Bench-to-bedside review: the importance of the precision of the reference technique in method comparison studies—with specific reference to the measurement of cardiac output. Crit Care.

